# Further Validation of the Short Form of the Self-Compassion Scale in a sample of Portuguese Medicine Students

**DOI:** 10.1192/j.eurpsy.2022.472

**Published:** 2022-09-01

**Authors:** F. Carvalho, A. Macedo, A. Manão, C. Cabacos, J. Azevedo, C. Marques, M. Marques, M. Carneiro, D. Telles Correia, F. Novais, C. Carvalho, A. Araújo, A.T. Pereira

**Affiliations:** 1 Faculty of Medicine of University of Coimbra, Institute Of Psychological Medicine, Coimbra, Portugal; 2 Centro Hospitalar e Universitário de Coimbra, Department Of Psychiatry, Coimbra, Portugal; 3 Coimbra Institute for Biomedical Imaging and Translational Research, Coimbra, Portugal; 4 Lisbon University, Faculty Of Medicine, Lisbon, Portugal; 5 University of Azores, Department Of Psychology, Ponta Delgada, Portugal

**Keywords:** validation, medicine students, self-compassion, SCS-SF

## Abstract

**Introduction:**

The Short Form of the Self-Compassion Scale (SCS-SF; Raes et al. 2011) is composed of 12 items that evaluate the same six dimensions (Self-Kindness/SK, Self-Judgement/SJ, Common Humanity/CH, Isolation, Mindfulness/M, Over-Identification/OI) as the long scale (26 items). The Portuguese version of the SCS-SF (Castilho et al. 2015) was validated in a vast sample from clinical and general populations, the latter being composed of students, other than from medicine courses.

**Objectives:**

To analyze the psychometric properties of the Portuguese version of the SCS-SF in a sample of Medicine/Dentistry students.

**Methods:**

Participants were 666 Portuguese medicine (82.6%) and dentistry (17.4%) students (81.8% girls); they answered an online survey including the SCS and other validated questionnaires from the OECD Study on Social and Emotional Skills/SSES: Stress resistance, Emotional control, Optimism and Persistence.

**Results:**

Confirmatory Factor Analysis showed that the model composed of six factors, two second order factors (positive and negative) and one third order factor (total) presented good fit indexes (χ2/df=3.013; RMSEA=.0066, p<.001; CFI=.970; TLI=.948, GFI=.947). The Cronbach’s alfas were .892, .869 and .877 respectively for the total, self-compassion and self-criticism dimension. Pearson correlations of the SCS-SF total score, self-compassion and self-criticism dimensional scores were moderate to high with the SSES measures, from .272/-.236/.247 with Persistence to .709/-.634/.615 with Optimism.

**Conclusions:**

Although reduced to less than half than the original SCS, the SCS–SF is a valid and useful alternative to measure general self-compassion and their positive and negative components in an ongoing longitudinal research with medicine/dentistry students.

**Disclosure:**

No significant relationships.

